# Shared Patterns of Gene Expression and Protein Evolution Associated with Adaptation to Desert Environments in Rodents

**DOI:** 10.1093/gbe/evac155

**Published:** 2022-10-21

**Authors:** Noëlle K J Bittner, Katya L Mack, Michael W Nachman

**Affiliations:** Department of Integrative Biology and Museum of Vertebrate Zoology, 3101 Valley Life Sciences Building, University of California Berkeley, California 94720; Department of Integrative Biology and Museum of Vertebrate Zoology, 3101 Valley Life Sciences Building, University of California Berkeley, California 94720; Department of Integrative Biology and Museum of Vertebrate Zoology, 3101 Valley Life Sciences Building, University of California Berkeley, California 94720

**Keywords:** adaptation, desert, RNA-Seq, genomics, rodents

## Abstract

Desert specialization has arisen multiple times across rodents and is often associated with a suite of convergent phenotypes, including modification of the kidneys to mitigate water loss. However, the extent to which phenotypic convergence in desert rodents is mirrored at the molecular level is unknown. Here, we sequenced kidney mRNA and assembled transcriptomes for three pairs of rodent species to search for shared differences in gene expression and amino acid sequence associated with adaptation to deserts. We conducted phylogenetically independent comparisons between a desert specialist and a non-desert relative in three families representing ∼70 million years of evolution. Overall, patterns of gene expression faithfully recapitulated the phylogeny of these six taxa providing a strong evolutionary signal in levels of mRNA abundance. We also found that 8.6% of all genes showed shared patterns of expression divergence between desert and non-desert taxa, much of which likely reflects convergent evolution, and representing more than expected by chance under a model of independent gene evolution. In addition to these shared changes, we observed many species-pair-specific changes in gene expression indicating that instances of adaptation to deserts include a combination of unique and shared changes. Patterns of protein evolution revealed a small number of genes showing evidence of positive selection, the majority of which did not show shared changes in gene expression. Overall, our results suggest that convergent changes in gene regulation play an important role in the complex trait of desert adaptation in rodents.

**Significance**

Finding genes underlying the convergent basis of evolution to extreme environments allows us to understand both how organisms have adapted to these environments and what paths are accessible to evolution. While most previous research has been limited to individual species, this study expands the scope by comparing RNA-Seq data from multiple rodent families to identify changes at the gene expression and protein level associated with adaptation to desert environments.

## Introduction

The repeatability of adaptive evolution at the molecular level remains an open question. In situations where the mutational target is small and constraints exist due to epistasis or pleiotropy, the molecular paths available to adaptation may be highly limited (Weinreich et al. 2006; Karageorgi et al. 2019). Indeed, there are a number of excellent examples of convergent molecular evolution underlying simple traits (e.g., Stewart and Wilson 1987; Mundy 2005; Zhen et al. 2012). For highly polygenic traits, however, convergence may be less expected simply because the mutational target is large and multiple paths may be available on which selection can act. Nonetheless, several studies have found evidence for convergence at the molecular level even for complex traits (e.g., Marcovitz et al. 2019; Sackton et al. 2019).

Convergent phenotypic evolution may be due to changes in gene regulation, to changes in protein structure, or both, yet these processes are rarely studied together in the context of complex adaptive traits (but see Hao et al. 2019). There is evidence that gene expression divergence and amino acid sequence divergence are correlated between paralogs following gene duplications (Gu et al. 2002; Makova and Li 2003), and more generally that rates of gene expression and rates of protein evolution are coupled in some lineages (e.g., Nuzhdin et al. 2004; Lemos et al. 2005). These observations raise the possibility that changes in both gene expression and protein sequence may contribute to the repeated evolution of complex adaptive traits.

Adaptation to desert environments in rodents provides an opportunity to study repeated evolution in both gene expression and protein sequence for a complex trait. Desert ecosystems present the challenge of extreme aridity and low or seasonally absent water, yet multiple lineages of rodents have independently evolved the ability to survive in these unusually harsh environments (reviewed in Degen 1997). Rodents have solved these challenges in myriad ways, including dietary specialization on plants that are high in water content or modifications to reduce evaporative water loss (Schmidt-Nielsen and Schmidt-Nielsen 1952; Schmidt-Nielsen 1964; Degen 1997). A common feature of most desert rodents is a modified kidney capable of producing highly concentrated urine (MacMillen and Lee 1967; Beuchat 1990; Al-kahtani et al. 2004; Donald and Pannabecker 2015). The production of hyperosmotic urine has evolved independently multiple times across rodents, and maximum urine concentration has been found to be correlated with environmental aridity in mammals (Rocha, Brito, et al. 2021). Final excreted urine concentration depends on the development and maintenance of a corticomedullary osmotic gradient within the kidney. Studies have shown that many aspects of kidney morphology and physiology have been modified in different lineages to produce hyperosmotic urine (Bankir and de Rouffignac 1985; Donald and Pannabecker 2015).

**Fig. 1:**
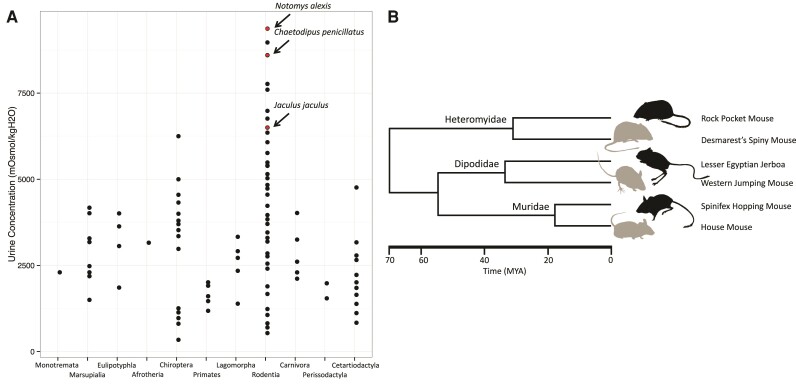
Species included in this study. (*A*) Estimates of urine concentration from across Mammalia from Beuchat (1990). Notably, Rodentia has representatives with the highest urine concentrations recorded in mammals. The three desert specialists in this study have among the highest urine concentrations measured in rodents. Note, while *Chaetodipus intermedius* has not been measured for urine concentration, *Chaetodipus penicillatus* is its sister taxon and is found in the same environment. (*B*) Phylogenetic relationships of target species: Rock Pocket Mouse (*C. intermedius*), Desmarest's Spiny Pocket Mouse (*Heteromys desmarestianus*), Lesser Egyptian Jerboa (*Jaculus jaculus*), Western Jumping Mouse (*Zapus princeps*), Australian Spinifex Hopping Mouse (*Notomys alexis*), and House Mouse (*Mus musculus*). Divergence time estimates from TimeTree.org. Illustrations by K. Mack.

The genetic basis of desert adaptation has been studied independently in a handful of species, and individual genes and pathways which may underlie this adaptive phenotype have been identified (Marra et al. 2012, 2014; Wu et al. 2014; MacManes 2017; Giorello et al. 2018; Tigano et al. 2020; reviewed in Rocha, Godinho, et al. 2021). Here, we study three phylogenetically independent lineages of rodents that have all converged on a common phenotype, ultra-high urine concentration associated with desert living, to identify shared molecular changes associated with habitat type. We compared gene expression and protein sequence divergence in the kidney, the organ responsible for sodium and water homeostasis, between a desert and a non-desert species in three comparisons representing transitions to desert living in three different rodent families (Heteromyidae, Dipodidae, and Muridae). Desert species were chosen based on their high urine concentration, a proxy for increased osmoregulatory capacity (fig. 1). Within Muridae, we compared the Australian Spinifex Hopping Mouse, *Notomys alexis*, the mammal with the highest known urine concentration and well studied for its modifications to desert life (MacMillen and Lee 1967; Macmillen and Lee 1969; Baudinette 1972; Donald et al. 2012), to the house mouse (*Mus musculus*), a widespread generalist. Within Dipodidae, we compared the desert-dwelling Lesser Egyptian Jerboa, *Jaculus jaculus*, previously studied for its kidney modifications associated with granivorous desert living (Schmidt-Nielsen and Schmidt-Nielsen 1952; Khalil and Tawfic 1963), to the Western Jumping Mouse, *Zapus princeps*, a North American species found in riparian environments. Within Heteromyidae, we compared the Rock Pocket Mouse, *Chaetodipus intermedius* (Bradley et al. 1975; Altschuler et al. 1979), native to the North American Sonoran desert, to the Desmarest's Spiny Pocket Mouse, *Heteromys desmarestianus*, a neotropical species found in mesic areas that cannot survive without free water (Fleming 1977).

We sequenced kidney mRNA from these pairs of taxa and assembled and annotated *de novo* transcriptomes for three desert-mesic species pairs spanning ∼70 million years of evolution. Assembled transcriptomes were used to analyze rates of evolution in single-copy orthologs to identify genes putatively under selection across desert lineages. We also performed mRNA sequencing on multiple individuals within each species to study gene expression divergence between desert and non-desert species. Global patterns of gene expression recapitulated the phylogeny of these six species. However, we also discovered a significantly greater number of shared changes in gene expression than expected by chance between desert and non-desert species. In contrast, shared changes in amino acid sequence were identified in a smaller proportion of genes.

## Results

### Sequencing, Assembly, and Annotation

We generated on average ∼123 million reads per sample for the assembly of *de novo* kidney transcriptomes in each species. For *M. musculus*, five smaller libraries were concatenated for assembly. After read correction, quality filtering, and adapter trimming, each library had an average of ∼103 million reads which were used for the assembly. Each assembly contained 965,227 transcripts on average. We reduced the number of redundant transcripts in the assembly to improve accuracy of downstream analyses by clustering similar transcripts together using CD-HIT-EST. This decreased the number of transcripts by ∼20% per sample to an average of 793,887 transcripts (supplementary table S2, Supplementary Material online). We used Benchmarking Universal Single-Copy Orthologs (BUSCO) to check assembly completeness to determine how many of the 6,192 orthologs found in the Euarchontoglires odb9 were present in our assembled transcriptomes. The six assemblies ranged in completeness from 80–87% (supplementary fig. S1, Supplementary Material online). This level of completeness reflects a single tissue (kidney) taken at one developmental time point. After open reading frame (ORF) prediction, we annotated each transcript to known *M. musculus* proteins. We were able to assign transcripts to 395,029 putative ortholog groups.

### Global Gene Expression Reflects Phylogenetic Relationships and Habitat Type

To identify patterns of differential gene expression, we sequenced kidney mRNA from additional individuals in each of the six species for an average of ∼27 million reads per individual. We retrieved 13,305 genes in *C. intermedius*, 11,749 genes in *H. desmarestianus*, 14,891 genes in *J. jaculus*, 14,380 genes in *Z. princeps*, 18,622 genes in *N. alexis*, and 19,913 genes in *M. musculus* for which we were able to quantify expression levels. These genes were annotated using *M. musculus* transcripts so, as expected, the number of genes we were able to annotate in more divergent species is more limited.

**Fig. 2:**
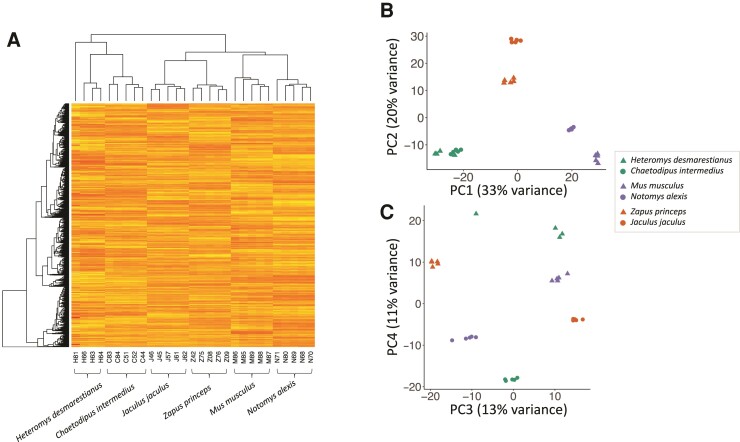
Expression-level variation differentiates species and habitat type. (*A*) Heat map showing relationships among samples based on gene expression clustering. With the exception of one sample (H81), expression patterns reflect phylogenetic relationships (see fig. 1). (*B*) Principal components (PC1 and PC2) for the expression data. PC1 explains 33% of the variance and reflects the phylogenetic relationships of the species. PC2 explains 20% of the variance. (*C*) Principal components (PC3 and PC4) for the expression data. PC4 explains 11% of the variance and differentiates samples by habitat type.

Gene expression profiles largely recapitulated the known phylogenetic relationships of these six species (fig. 2*A*). Individuals within each species form well-defined clusters (apart from a single *H. desmarestianus* individual), and the different genera within each family share expression profiles that are more similar to each other than they are to genera in different families. Further, Muridae and Dipodidae are more similar to each other in expression profiles than either is to Heteromyidae, reflecting the known evolutionary relationships of these families. Thus, the overall expression patterns reflect evolutionary history more than habitat type. These patterns are also seen in a principal component analysis (PCA) based on expression-level co-variance (fig. 2*B*), where PC1 (accounting for 33% of the variance) largely reflects phylogeny. Despite the overall phylogenetic pattern of gene expression, consistent differences in expression were seen between desert and non-desert species within each family. In particular, PC4 captures this variation, separating desert from non-desert taxa (explaining 11% of the variation; fig. 2*C*).

### Shared Patterns of Differential Expression in Desert Rodent Kidneys

We quantified differential expression (DE) between desert and non-desert species within each family. In pairwise contrasts between desert and non-desert species in Heteromyidae, Dipodidae, and Muridae, we identified >4,000 genes in each comparison with evidence of significant DE (supplementary table S3, Supplementary Material online, FDR < 0.01). DE between desert and non-desert species in each of the three families was associated with several GO categories, including cellular metabolic processes and nitrogen metabolic processes (supplementary table S4, Supplementary Material online). We identified a total of 654 genes that showed significant DE in all three species pairs (supplementary fig. S2, Supplementary Material online), with 145 of these genes showing shifts in the same direction in each comparison.

**Fig. 3:**
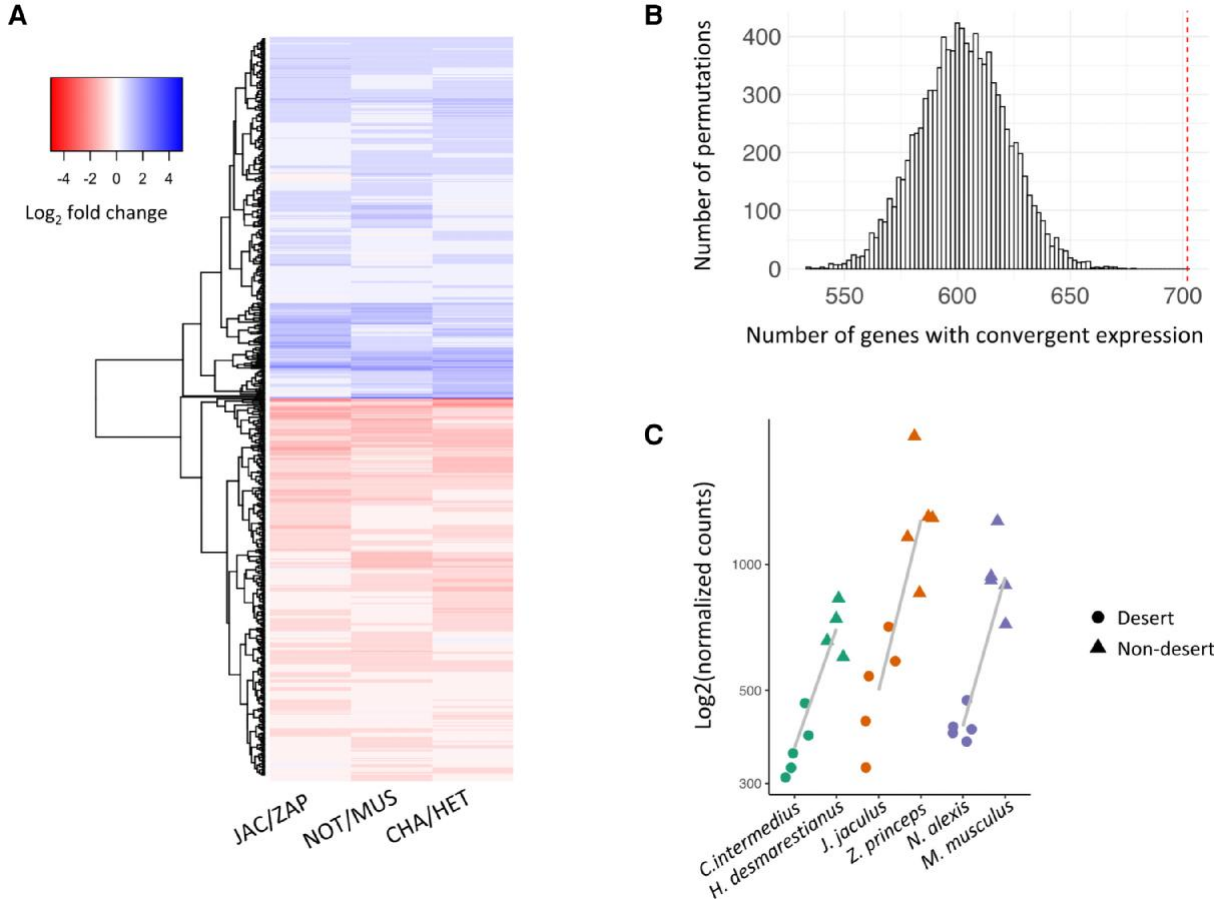
Shared patterns of gene expression. (*A*) Heatmap of the 702 genes with evidence of shared gene expression patterns. Each row is a gene. Each of the three columns shows the mean expression value among all desert individuals compared to the mean expression value of all non-desert individuals for each family. (*B*) Number of genes expected by chance to show shared expression after 10,000 permutations. Observed number of genes (dashed vertical line) is greater than the distribution expected by chance (*P* < 0.0001). (*C*) Expression values for *Aqp11*, a gene showing shared patterns of gene expression. In all comparisons, desert species show lower expression levels compared with their non-desert relative. Lines are drawn between the mean of normalized expression for each desert-mesic pair. Points are jittered for clarity.

To identify shared shifts in gene expression associated with desert living, we also modeled gene expression as a function of species pair (i.e., family), habitat, and their interaction. Shared changes were identified as those for which there was a significant effect of habitat (FDR < 0.01), but no interaction between species pair and habitat (FDR > 0.05; see Materials and Methods; Parker et al. 2019). We identified 702 genes with shared shifts in desert rodents relative to the mesic comparison (fig. 3*A*). This set includes all the 145 genes identified above in pairwise tests. Thus, 8.6% (702/8,174) of genes showed shared shifts in expression in desert rodents compared with their non-desert relatives.

Shared shifts in gene expression can be a consequence of selection in response to shared environmental pressures or stochastic processes. These shared environmental pressures are not limited to aridity but may extend to other differences in habitat type, diet, condition, and more. To ask if the observed number of genes with shared DE was more than expected by chance, we performed a permutation test in which we took each gene and randomly switched habitat assignment within species pairs, while always maintaining the same label for all biological replicates within a species, to create 10,000 permuted data sets (fig. 3*B*; see Materials and Methods; Parker et al. 2019). Permuted data sets never identified more genes than the observed set of shared genes, suggesting an enrichment of shared DE associated with habitat type under the simplifying assumption that expression changes at genes are independent (see Discussion).

Shared changes in gene expression could be due to convergent evolution or to a similar plastic response in similar xeric environments. To disentangle these, we analyzed RNA-seq data from a previous study in which *M. musculus* from a relatively mesic environment in Canada were subjected to three days of water deprivation in the laboratory (Bittner et al. 2021). We compared the identity of genes showing a plastic response in that study to the identity of genes showing an evolved response between *N. alexis* and *M. musculus* in the present study. In the former study, 1,354 genes were differentially expressed between control and treatment (water deprivation) groups, while in the present study, 8,008 genes were differentially expressed between *N. alexis* and *M. musculus*. The overlap between these sets was 547 genes, and this overlap is not more than expected by chance (hypergeometric test, *P* = 0.99). This suggests that the vast majority of differentially expressed genes (7461/8008 = 93%) between *N. alexis* and *M. musculus* reflect evolved differences rather than plastic responses to xeric conditions. We also compared the 1,354 genes showing a plastic response in Bittner et al. (2021) with the 145 genes in this study in which all three species pairs showed expression changes in the same direction. The overlap was nine genes, and this is not more than expected by chance (hypergeometric test, *P* = 0.99). Thus, the majority of genes showing shared shifts in gene expression in this study ([145 − 9]/145 = 94%) do not show a plastic response to water stress in *M. musculus* under laboratory conditions. From these analyses, we argue that most shared changes in gene expression reflect convergent evolution rather than phenotypic plasticity.

The magnitude of expression differences between individual desert-mesic pairs was often modest in one or more contrasts between species pairs (supplementary fig. S3, Supplementary Material online); only 208 genes with shared expression shifts showed an average of greater >0.5 log_2_-fold change difference between each desert-mesic species pair. The number of genes showing higher expression in desert rodents compared with non-desert relatives (335 genes, shown in blue in fig. 3*A*) was slightly fewer than the number of genes showing lower expression in desert rodents compared with non-desert relatives (367 genes, shown in red in fig. 3*A*). Additionally, across all genes, fold changes between individual desert-mesic species were found to be significantly correlated in two of the three comparisons of species pairs (Spearman's rank correlation *rho*, *C. intermedius/H. desmarestianus* vs*. J. jaculus/Z. princeps*, *P* = 0.0062, *rho* = 0.03; *C. intermedius/H. desmarestianus* vs. *N. alexis/M. musculus*, *P* < 2.2e-16, *rho* = 0.10; *N. alexis/M. musculus* vs*. J. jaculus/Z. Princeps, P* = 0.12, *rho* = −0.017).

To identify genes and pathways of interest, we divided the shared set of differentially expressed genes into those that are upregulated with respect to the desert taxa in all comparisons and those that are downregulated with respect to the desert taxa in all comparisons and performed phenotype and gene ontology (GO) term enrichment tests on these (see Materials and Methods). Genes upregulated across desert rodents were enriched for several GO terms related to gene regulation, including regulation of RNA metabolic process (*q* = 2.55 × 10^−5^), regulation of gene expression (*q* = 1.34 x 10^−5^), and regulation of RNA biosynthetic process (*q* = 3.87 × 10^−5^). Genes downregulated in desert rodents were enriched for GO terms related to metabolic processes, including metabolic process (*q* = 1.56 × 10^−3^), organic substance metabolic process (*q* = 3.93 × 10^−3^), and cellular metabolic process (*q* = 3.54 × 10^−3^). Genes with shared patterns of expression included those with mouse mutant phenotypes related to kidney development and physiology or homeostasis (supplementary table S5, Supplementary Material online). For example, Aquaporin 11 (*Aqp11*) is expressed at a lower level in all desert species compared with non-desert species in all three comparisons (fig. 3*C*). This gene is part of a family of genes encoding membrane-integrated channels responsible for water transfer across membranes throughout the body. This set also includes genes associated with human phenotypes related to kidney and renal diseases (supplementary table S6, Supplementary Material online); for example, mutations in the gene *Col4a5*, which is downregulated in desert species, have been associated with Alport syndrome, a disease characterized by kidney inflammation (Köhler et al. 2019).

### Genes Under Selection in Desert Lineages

Next, we tested for evidence of selection on protein-coding sequences using well-aligned single-copy orthologs found in all desert-mesic species pairs. We performed three analyses using the 1,474 single-copy orthologs with high-quality alignments present in all species. First, we searched for genes showing signatures of selection shared by all desert species by calculating the ratio of non-synonymous substitutions per non-synonymous site to synonymous substitutions per synonymous site (ω) at each locus to estimate rates of evolution along each branch implemented using a branch model for codeml in PAML (Yang, 1998, 2007). When testing for evidence of selection shared only by desert species compared with non-desert species, we uncovered 39 genes (39/1,474 = 2.6%) for which ω was greater in all three desert lineages (supplementary table S7, Supplementary Material online, FDR < 0.1). This group of genes is enriched for phenotypes related to multiple aspects of the immune response as well as to hearing/vestibular/ear phenotypes and other aspects of osteology (supplementary table S8, Supplementary Material online). Immune genes are some of the fastest evolving genes in the genome and are disproportionately found to be under selection in many studies (Hurst and Smith 1999; Schlenke and Begun 2003; Nielsen et al. 2005). One gene of particular interest is FAT atypical cadherin 4 (*FAT4*) (*q* = 0.018). *FAT4* has been implicated in human kidney diseases (Alders et al. 2014) and is involved in normal kidney development through modulating the RET signaling pathway in mouse models (Mao et al. 2015; Zhang et al. 2019). *FAT4* homozygous knockout mice have smaller kidneys with cysts in renal tubules when compared with wild-type mice and die within a few hours of birth (Saburi et al. 2008). These phenotypes in laboratory mice make this an interesting candidate gene for future studies in desert rodents. We found three genes that showed evidence of positive selection and also showed shared shifts in gene expression (Rows 1–3 in supplementary table S9, Supplementary Material online); however, they are not known to be associated with phenotypes of interest. This amount of overlap is no more than expected by chance (hypergeometric test, *P* = 0.64).

We then tested for lineage-specific selection by allowing two values of ω on the tree. We calculated ω for each of the three desert lineages individually and compared this to the value of ω for the five remaining taxa for each ortholog. This analysis was implemented as above but restricts the comparison to one species instead of all three. We identified 23 genes in *C. intermedius*, 19 in *J. jaculus*, and 18 in *N. alexis* where ω was significantly elevated (at FDR <0.1; supplementary table S10, Supplementary Material online) compared with all other taxa. These genes are candidates for underlying lineage-specific adaptations. In *C. intermedius*, enriched phenotypes were related to immunity and morphological traits including kidney size, while in *J. jaculus* and *N. alexis*, enriched phenotypes were related to behavioral and electrophysiological traits (supplementary table S11, Supplementary Material online). In the *C. intermedius* comparison, *Dusp4* is of some interest as it has been associated with aberrant circulating solute levels in mouse models. Deletion of this gene has been associated with increased excreted protein and altered kidney structure in diabetic mice (Denhez et al. 2019). It was also found in the set of genes showing shared DE. Overall, the amount of overlap (hypergeometric test, *P* > 0.06 in all comparisons) between any of these lists and differentially expressed genes between lineage pairs is no more than expected by chance (supplementary table S9, Supplementary Material online).

In the third analysis, we employed a branch-site model to identify genes in which specific codons may be under positive selection. In this approach, genes for which specific codons have a ω > 1 in the “foreground” branches (defined to include all three desert species) compared with the “background” branches are identified. Seven genes were identified (supplementary table S12, Supplementary Material online) with codons under selection in all three desert lineages, including *Coro2b*, a gene implicated in abnormal renal glomerulus morphology (Schwarz et al. 2019) and urine protein level (Rogg et al. 2017) and *Bloc1s4*, which is implicated in abnormal renal physiology (Gwynn et al. 2000). Again, there was no significant overlap with the genes identified in the DE analysis (*P* = 0.47; supplementary table S9, Supplementary Material online).

## Discussion

The molecular basis of convergent evolution has been well studied for a number of simple traits but has been less studied for complex traits. Even fewer studies have compared convergence in both gene expression and protein evolution for complex traits. Here, we studied shared changes in gene expression and amino acid sequence in three species of desert rodents and their non-desert relatives from across the rodent tree, representing ∼70 million years of evolution to identify candidate genes for convergent evolution underlying this complex adaptive trait.

Despite the long evolutionary timeframe and the fact that most expression patterns tracked phylogeny (fig. 2), we identified hundreds of genes (702/8,174 = 8.6%) that showed shared shifts in gene expression (fig. 3*A*). This number is more than expected by chance under a model of independent gene evolution (fig. 3*B*). It is important to recognize that the number of genes showing shared expression changes does not reflect the number of causative changes (i.e., mutational events in evolution), as many of these shared changes in expression might reflect downstream consequences of a smaller number of changes at upstream regulators that govern networks of co-regulated genes. Nonetheless, the large number of shared changes in expression suggests that a measurable amount of desert adaptation is mediated by a large set of shared changes in gene regulation, whether at the level of individual genes or through sets of co-regulated genes. Future studies aimed at understanding the precise mechanisms mediating expression levels in each lineage would help quantify the degree to which these patterns are the result of *cis*-regulatory changes at the same locus or shared upstream *trans*-regulators that mediate shifts in expression across sets of co-regulated genes.

Our analysis identified specific genes of interest known to be involved in osmoregulation and kidney function. Of note, *Apq11* was found to be expressed at lower levels in all desert taxa compared with their non-desert counterparts. Aquaporins have been repeatedly implicated in studies of desert adaptation across rodents (Marra et al. 2012, 2014; Pannabecker 2015; Giorello et al. 2018). Mouse knockouts have demonstrated that *Aqp11* is necessary for proximal tubular function and the formation of healthy kidneys (Morishita et al. 2005; Tchekneva et al. 2008). In addition, *Aqp11* plays a role in salivary gland development (Larsen et al. 2010). This paper provides further evidence that aquaporins are a common evolutionary target in desert adaptation.

In addition to these shared changes in gene expression, we identified a large number of species-specific changes in each species pair. Perhaps not surprising given the long evolutionary timescales and complexity of osmoregulatory function, much of the evolutionary response appears to be specific to individual lineages.

In principle, phenotypic plasticity could also mediate some of the observed expression differences as most of the animals were caught in the wild and therefore experienced different environments, presumably with differences in hydration status. However, several observations suggest that evolved changes, rather than plastic changes, underlie most of the observed expression differences. First, comparison of the plastic expression response of *M. musculus* when deprived of water (Bittner et al. 2021) to the expression differences observed between *M. musculus* and *N. alexis* (this paper) revealed little gene overlap, suggesting that >93% of the differences seen between *M. musculus* and *N. alexis* reflect evolved changes. Second, one of the desert-adapted species (*J. jaculus*) was from a laboratory colony in which animals were reared with free access to fresh vegetables. Comparisons between *J. jaculus* and *Z. princeps* identified many differentially expressed genes, including many that were shared among the other comparisons, indicating that most expression divergence does not reflect expression plasticity due to differences in recent water availability. Third, apple was offered to most of the wild-caught species for a period before they were sacrificed, mitigating the effects of short-term water stress. Fourth, many of the desert-adapted species will not accept free water if offered. Finally, we note that the overall patterns of expression (fig. 2) recapitulate the known phylogeny of these species (fig. 1), consistent with the idea that most expression variation is due to evolved differences. While not discounting that some of the observed differences in expression may reflect phenotypic plasticity, these observations strongly support the inference that most shared changes are due to convergent evolution.

In contrast to the fairly long evolutionary timescales in the present study, we documented differences in kidney gene expression between desert and non-desert populations of *M. musculus* separated by only a few hundred generations of evolution (Bittner et al. 2021). In that study, we identified 3,935 differentially expressed genes of which 99 were found in the list of genes with shared DE patterns in all three desert lineages in the present study. The lack of significant overlap (hypergeometric test, *P* = 0.99) suggests that over long evolutionary timescales, adaptive responses to xeric conditions may be different from the evolved changes in gene expression over short evolutionary timescales.

In contrast to the number of shared changes in gene expression, we observed few genes that showed evidence of positive selection on amino acid sequences (39/1,474 = 2.6%) among desert species. These proportions are not directly comparable, as the methods used to detect shared expression changes and positive selection are quite different. Nonetheless, our analyses suggest that the phenotypic convergence seen in urine concentration is reflected at the molecular level more in patterns of gene regulation than in patterns of protein evolution. In this study, we took advantage of RNA-seq data to also study protein evolution in non-model organisms whose genomes have not yet been sequenced. However, this approach has limitations. By restricting our analyses to single-copy orthologs found in all transcriptomes, we were only able to survey a small proportion of the protein-coding genes that are known to be expressed in the kidneys. Future analyses based on whole-genome sequences would provide a clearer picture of protein evolution.

Although expression evolution and amino acid sequence evolution have been found to be correlated in some cases (Nuzhdin et al. 2004; Lemos et al. 2005), we did not find significant overlap in the number of genes showing shared patterns of gene expression and shared signatures of selection. The small amount of overlap might reflect differences in the selection pressures on these two classes of changes. For example, *cis*-regulatory changes in gene expression are often controlled in a tissue-specific and developmental-stage-specific manner, and as such are expected to be less pleiotropic and thus less constrained in evolution (e.g., Wray 2007). Protein-coding changes, on the other hand, affect all tissues and developmental stages in which the protein is expressed and thus may be more pleiotropic and consequently more constrained. The small amount of overlap might also reflect both statistical and methodological limitations of our study. First, the analytic methods used to detect shared expression changes and shared signals of selection are quite distinct and likely have different false-negative and false-positive rates. Second, we studied gene expression in adults, yet gene expression varies considerably during kidney development (Schwab et al. 2003) and early expression is undoubtedly important in establishing morphological differences between desert and non-desert kidneys. Third, kidneys have a heterogenous cellular composition, and changes in cellular composition between species are likely to affect measures of gene expression in bulk preparations. Future studies of gene expression in single cell preparations at early developmental time points might uncover additional signals of expression variation associated with desert conditions. It would also be valuable to study expression changes in both males and females as the demands of water balance may be especially acute in lactating females.

Despite these caveats, we identified a number of potential candidate genes associated with desert living, including some that showed both shared gene expression and shared amino acid sequence evolution. The target available to selection in a trait as complex as desert adaptation is likely large and constrained along each lineage to a different degree by other aspects of the organism's morphology and physiology. Nonetheless, an interesting outcome of our analysis is that a number of the genes and pathways identified here are similar to those identified in other studies of rodent and mammalian desert adaptation (Marra et al. 2012, 2014; Wu et al. 2014; MacManes 2017; Giorello et al. 2018; Tigano et al. 2020). It is clear that gene families such as aquaporins, which are responsible for facilitating water transport across membranes, and solute carriers, may play a role in mitigating water loss across multiple systems and therefore underlie convergent evolution at the genetic level to desert environments.

Altogether, our results demonstrate the power of gene expression studies in a phylogenetically controlled manner for identifying shared changes associated with habitat. Incorporating whole-genome (or whole exome) sequences of these and other taxa could provide a more complete comparison of protein coding and gene expression changes in facilitating adaptation to deserts.

## Materials and Methods

### Sample Collection

Five adult male mice for each species, apart from *H*. *desmarestianus*, for which only four samples could be obtained, were included in this study. *Chaetodipus intermedius*, *Zapus princeps*, and *Mus musculus* were caught by N. Bittner using Sherman live traps set overnight following the guidelines of the American Society of Mammalogists (Sikes and Gannon 2011) and an ACUC protocol approved by UC Berkeley (AUP-2016-03-8536). Both desert and non-desert animals were offered apple after capture to avoid acute dehydration in traps. Mice were euthanized by cervical dislocation, and kidney and liver were removed and preserved in RNAlater according to manufacturer's instructions. *C. intermedius* were trapped near Tucson, AZ, USA, *Z. princeps* were trapped at Sagehen Creek Field Station near Truckee, CA, USA, and *M. musculus* were trapped near Berkeley, CA, USA. *Heteromys desmarestianus* were collected in Chiapas, Mexico by Beatriz Jimenez, and *Notomys alexis* were collected by Kevin Rowe in Northern Territory, Australia. Mice collected by N. Bittner were prepared as museum specimens (skins and skulls) and deposited in the collections of the UC Berkeley Museum of Vertebrate Zoology. Animals collected by K. Rowe were prepared as museum specimens and deposited at Museums Victoria. The collecting localities, collector's numbers, and museum catalog numbers for each specimen are provided for all wild-caught animals in supplementary table S1, Supplementary Material online. Samples from *J. jaculus* were provided by Kim Cooper at UC San Diego from an outbred lab colony.

### mRNA Library Preparation and Sequencing

To target loci underlying adaptation to desert environments, we focused on genes expressed in the kidney. RNA was extracted from kidney preserved in RNAlater using the MoBio Laboratories Powerlyzer Ultraclean Tissue & Cells RNA Isolation Kit. Remaining DNA was removed with DNAse-1 followed by a Zymo RNA Clean and Concentrator column clean-up. Due to the lower quality of some samples (RIN scores <5), a ribosomal RNA depletion step was performed with a KAPA Riboerase Kit before libraries were prepared with the KAPA HyperPrep Kit. Libraries were pooled and sequenced across two lanes of 150 bp PE Illumina NovaSeq (one lane of S1 and one of SP) at the Vincent J. Coates Genomics Sequencing Center at UC Berkeley. One library from each species (except *M. musculus*; see below) was sequenced at greater depth for transcriptome assembly; these were sequenced to a target of 100M read pairs while the remaining 24 libraries, intended for expression analysis, were sequenced to a target of 20M read pairs (see supplementary file S1, Supplementary Material online).

### Transcriptome Assembly

For each of the five 100M-read-pair libraries, reads were examined for quality metrics with FastQC (https://www.bioinformatics.babraham.ac.uk/projects/fastqc/) and then corrected by removing erroneous k-mers using rCorrector (Song and Florea 2015). Adapters and poor-quality sequence were trimmed using Trim Galore! (https://www.bioinformatics.babraham.ac.uk/projects/trim_galore/). As FastQC revealed a large quantity of duplicates within the sequenced libraries, which is likely in part due to rRNA contamination, we chose to remove all reads that mapped to known rodent rRNA from NCBI using bowtie2 (Langmead and Salzberg 2012). We ran Trinity v2.1.1 (Grabherr et al. 2011) to generate a transcriptome assembly for each species. Because transcriptome-depth (i.e., 100M read pairs) sequencing was not done for *M. musculus*, reads from all five individuals (approximately equal to the sequencing depth for transcriptome individuals) were combined to assemble the transcriptome of a local individual as above (supplementary table S2, Supplementary Material online). To remove redundant transcripts from the Trinity assembly, transcripts with ≥95% sequence identity were clustered with CD-HIT-EST (settings: -c 0.95 -n 8; Li and Godzik 2006) to create representative transcripts before use in downstream analysis (supplementary table S2, Supplementary Material online). This was done to collapse transcript isoforms as well as to remove transcripts created by assembly errors (chimeras, duplicates, misassembled transcripts, and the like). Transrate (Smith-Unna et al. 2016) was used to calculate assembly statistics. To assess assembly completeness, we used BUSCO (Manni et al. 2021) to look for the 6,192 orthologs found in the Euarchontoglires odb9 database and thus expected to exist in the taxa studied here.

### Transcriptome Annotation and Ortholog Detection

To identify coding regions within our assembled transcripts for downstream analyses, we utilized TransDecoder v5.5.0 (http://transdecoder.sourceforge.net). We identified the longest ORF and searched for matches to both the Pfam protein domain database (Bateman et al. 2004) and mouse-specific SwissProt database (Bairoch and Apweiler 2000) to retain ORFs based on homology. As high-quality gene annotations were available for *M. musculus*, we used the curated RefSeq protein database for this species. Orthologous gene groups across all six taxa were identified using OrthoFinder v2.3.3 (setting: -S diamond; Emms and Kelly 2015). To minimize the number of alternate isoforms used in the analysis, we only used the longest ORF identified per gene.

### mRNA Read Mapping

Raw reads from all libraries were examined for quality with FastQC. Adapters and poor-quality sequence were trimmed using Trimmomatic v0.36 (Bolger et al. 2014). The five libraries that were generated for transcriptome assembly were subsampled to the average read number of the libraries generated for expression (27,787,405 reads). Reads were mapped to transcriptomes generated for each species with Salmon v 0.14.1 (Patro et al. 2017). To compare across genera, transcripts were annotated using BLASTn to the RefSeq cdna database for *M. musculus*. Read counts were summed across transcripts for each annotated gene.

### Quantification of Gene Expression and Identification of Shared Patterns of Differential Expression

DESeq2 (Love et al. 2014) was used to normalize for differences in library size and to call DE between species within each family and across all samples. As transcripts between species can differ in length, a length correction was applied. Reads were subsequently transformed with a variance stabilizing transformation for PCA.

We used DESeq2 to identify shared patterns of DE between desert and non-desert species across all three families using an approach similar to that used by Parker et al. (2019). We fit a generalized linear model for gene expression as a function of habitat (desert vs. non-desert), family (species pair), and their interaction. Genes were classified as shared and differentially expressed in cases where there was a significant effect of habitat (desert vs. non-desert, FDR < 0.01) but no interaction effect of species pair by habitat (FDR > 0.05). This analysis was restricted to genes with greater than an average of 20 reads per sample for each species, resulting in a total of 8,174 genes. *P*-values were adjusted for multiple testing using a Benjamini and Hochberg (1995) correction. Adjusted *P*-values (FDR) are reported in the results. DE within each species pair was identified using pairwise contrasts. For pairwise contrasts, genes with a mean of fewer than ten reads per sample were removed from the analysis.

Shared expression differences among pairs of desert and non-desert species could be due to convergent evolution in response to similar selection pressures, a similar plastic expression response in similar environments, or chance. To assess the contribution of phenotypic plasticity to observed expression differences between species, we analyzed a previously published RNA-seq data set from *M. musculus* in which mice were deprived of water for three days (Bittner et al. 2021). Comparisons between control mice (water *ad libitum*) and treatment mice (water deprived) were used to identify expression changes in the kidney that reflect plastic responses to xeric conditions. This set of genes was then compared with the set of genes showing expression divergence between *M. musculus* and *N. alexis* and with the set of genes showing expression divergence across all three xeric-mesic species pairs. These analyses suggest that only a small fraction of the expression differences between species is due to phenotypic plasticity (see Results). In addition, we note that one desert-adapted species (*J. jaculus*) was reared in the laboratory with access to water. Therefore, expression differences between *J. jaculus* and *Z. princeps* cannot be attributed to differences in the availability of water.

To assess whether more genes showed shared DE shifts by habitat type than expected by chance, we used permutation tests as described in Parker et al. (2019). For each gene, read counts were randomly assigned to habitat within each species pair. All biological replicates (i.e., all five individuals) in each species were assigned to the same habitat. This process was used to create 10,000 permuted data sets. The number of shared differentially expressed genes in these data sets was compared with that of the observed data set. We note that this approach, while used in other studies, treats genes as independent and so may not fully account for shared expression responses in sets of co-regulated genes.

### Estimating Rates of Molecular Evolution and Identification of Genes Under Positive Selection

Using the single-copy ortholog groups generated by OrthoFinder for all six species, we aligned these using Guidance2 (Sela et al. 2015) with the standard parameters for a MAAFT alignment for amino acids. This provided alignment quality scores for all 1,855 genes and we removed those for which the alignment quality score was poor (mean column score <0.8) resulting in a set of 1,474 genes with protein-coding alignments for subsequent analyses. This reduced data set reflects the fact that to be included, a gene must be expressed and well assembled in the transcriptomes of all six species.

To identify genes in desert lineages displaying evidence of selection, we calculated the ratio of nonsynonymous substitutions per nonsynonymous site to synonymous substitutions per synonymous site (ω) at each locus to estimate rates of evolution along each branch. We used a maximum likelihood approach in a phylogenetic context by implementing the codeml package in PAML v 4.9 (Yang 1997). The following three analyses were implemented using alignments for the 1,474 single-copy orthologs with high-quality alignments present in all species with an unrooted species tree: (*M. musculus*, *N. alexis*), (*Z. princeps*, *J. jaculus*), (*H. desmarestianus*, *C. intermedius*).

In the first analysis, we tested for evidence of selection shared by and exclusive to all three desert lineages for each ortholog. We reason that genes with elevated rates of evolution in organisms with shared habitat types may relate to adaptation to that environment. To this end, we used a two-ratio branch model (model 2) in which we allow ω to vary along the branches using the F3 × 4 codon model. For each gene, we estimated ω along the three desert branches and estimated a separate value for ω along the three non-desert branches (Yang 1998, 2007). While traditionally a value of ω > 1 is considered evidence of positive selection, this is generally considered too strict for a gene-wide analysis, as selection is thought to be acting on individual codons rather than on the entire gene. Therefore, a significantly greater value of ω in desert lineages compared with non-desert lineages is considered evidence of positive selection, while the converse is considered evidence of purifying selection. A greater value of ω in desert lineages compared to non-desert lineages could also be caused by a relaxation of selective constraint in desert lineages. Significance is measured by comparing the value with a null model using a χ^2^ test. *P*-values were adjusted for multiple testing using a Benjamini and Hochberg (1995) correction. These adjusted *P*-values (FDR) are reported. This analysis was intended to identify genes underlying desert adaptation common to all three species.

In the second analysis, we utilized the framework as in the first analysis but executed it for each desert species individually. We used a two-branch ratio model and compared *C. intermedius*, *J. jaculus*, and *N. alexis* individually to the other five species in the tree. This analysis was intended to identify species-specific adaptations. We hypothesize that while genes under selection in all three desert lineages are intriguing candidates for desert selection, many lineage-specific modifications may contribute to adaptation to extreme environments. Values of ω were compared at each locus with a null model as above to assess for evidence of selection.

For the third analysis, we performed a branch-site model which allows ω to vary both across sites in the protein and across branches on the tree to allow detection of specific codons under selection. The first two analyses (above) calculate averages of locus-wide estimates of ω. By interrogating each codon, a more localized signal of selection may be identified. For this analysis, we tested codons for values of ω > 1 along branches for multiple values of Ω (0.1, 0.5, 1.5) and compared this to a null model, as described above.

### Enrichment Analyses

For gene sets of interest, GO category enrichment tests were performed with GOrilla (Eden et al. 2009) implemented with their online platform. For the sequence-based analyses, we compared the subset of genes with evidence of selection against the remainder of genes used in the analysis to determine whether the subset was enriched for GO categories of interest. We retained those with an FDR corrected *P*-value generated by GOrilla <0.05 for further analysis. For the expression-level analyses, we compared all significant genes regardless of direction of expression difference against the remainder of genes for which we had expression information. Phenotype enrichment tests were performed with modPhea with their online platform (Weng and Liao 2017) using the same framework.

## Supplementary Material

evac155_Supplementary_Data

## Data Availability

Illumina sequencing data from this study are available through the NCBI Sequence Read Archive under accession PRJNA656179. Samples collected by N.K.J.B. are accessioned into the Museum of Vertebrate Zoology collection.
