# Supplementary material for: Shared Patterns of Gene Expression and Protein Evolution Associated with Adaptation to Desert Environments in Rodents
Source: Genome Biol Evol. 2022 Oct 21;14(11):evac155. doi: 10.1093/gbe/evac155 (PMC9648513; doi:10.1093/gbe/evac155)

Supplemental material

Figure S1. Benchmarking Using Single Copy Orthologs (BUSCO) score for each of the transcriptome assemblies.

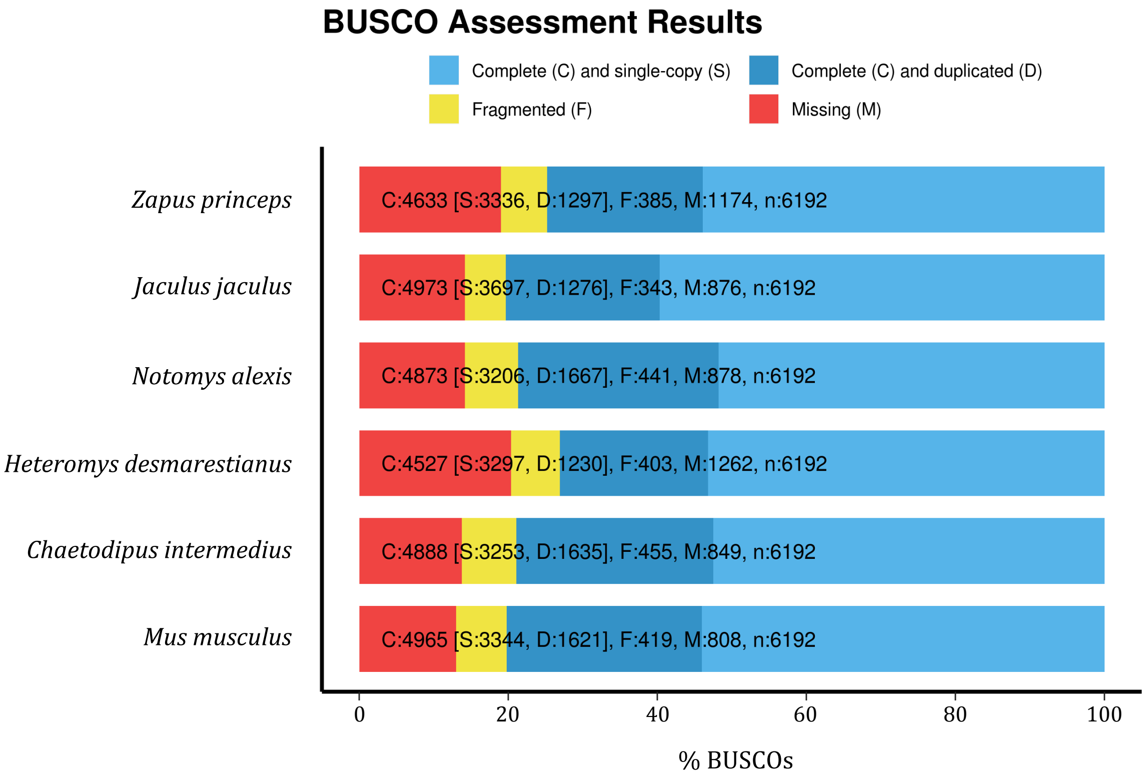

Figure S2. Genes that are differentially expressed between each desert and non-desert comparisons within each family and the overlaps among families.

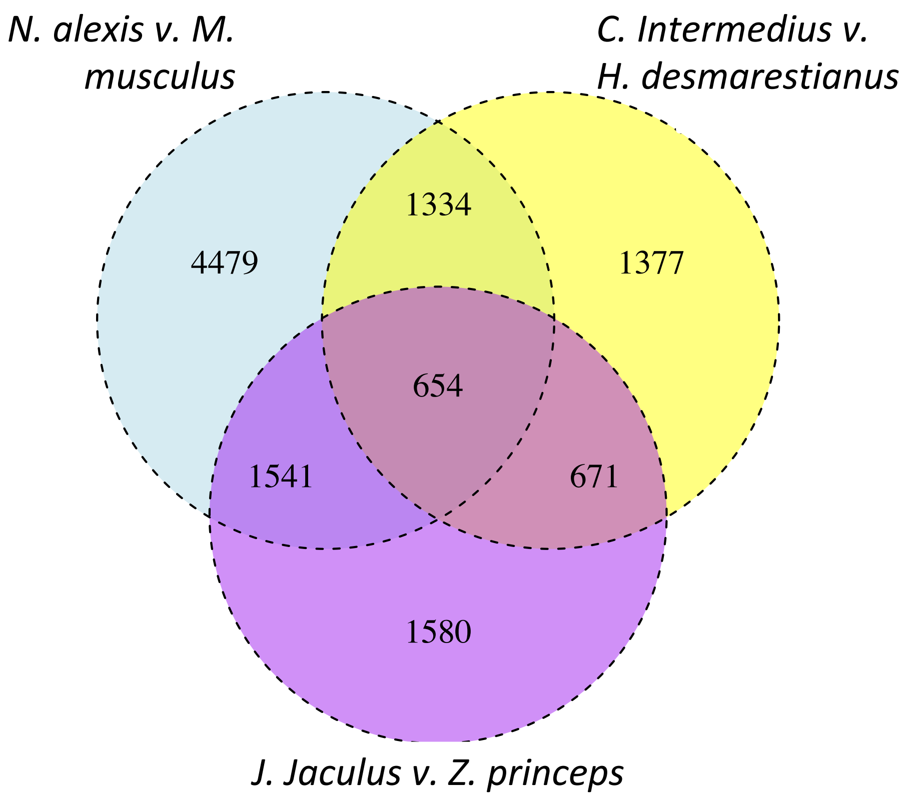

Figure S3. Magnitude of expression differences between each desert-mesic species pair.

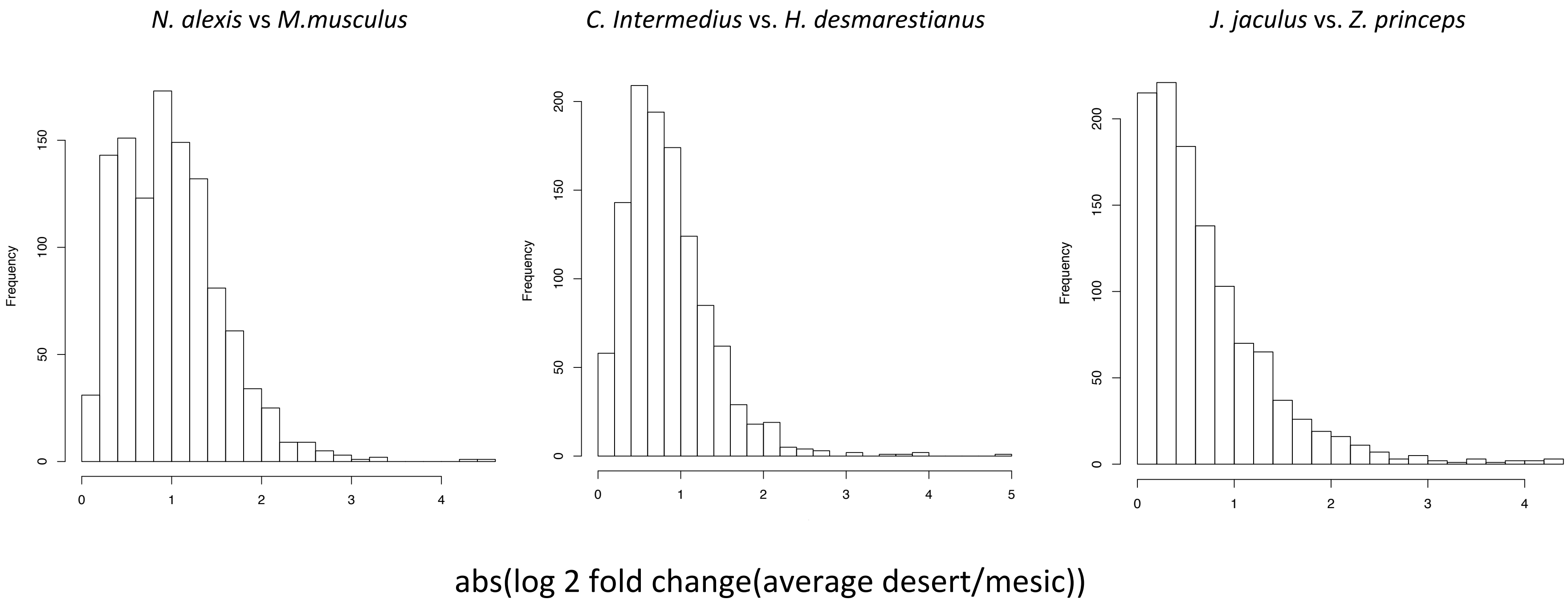

Supplement: evac155_Supplementary_Data [file evac155_supplementary_data.zip › Bittner et al_supplementalfig_Oct28.pdf]
